# Pre-injection of Zebrafish (*Danio rerio*) *tnfb* Polyclonal Antibody Decreases the Mortality of *Vibrio vulnificus* Infected Zebrafish

**DOI:** 10.3389/fvets.2021.741242

**Published:** 2021-11-18

**Authors:** Suyi Li, Cong Jiang, Hua Chen, Lijuan Zhang, Ling Ke, Xu Chen, Chentao Lin

**Affiliations:** ^1^Institute of Biotechnology, Fujian Academy of Agricultural Sciences, Fuzhou, China; ^2^Fujian Key Laboratory of Marine Enzyme Engineering, College of Biological Sciences and Engineering, Fuzhou University, Fuzhou, China; ^3^The Public Service Platform for Industrialization Development Technology of Marine Biological Medicine and Product of State Oceanic Administration, Fujian Normal University, Fuzhou, China; ^4^Center of Engineering Technology Research for Microalgae Germplasm Improvement of Fujian, Southern Institute of Oceanography, Fujian Normal University, Fuzhou, China

**Keywords:** *tnfb*, polyclonal antibody, *Vibrio vulnificus*, immunity, zebrafish

## Abstract

Tumor necrosis factor (TNF) plays an important role in an inflammatory cytokine storm. Over-secretion of TNF by the host in response to infection aggravates the disease. TNF expression level is positively correlated with the mortality caused by some bacterial infections. Therefore, using TNF antibody may alleviate the inflammation to resist bacterial infections. The function of fish TNF-b antibody in bacterial infection is still unclear. In this study, infection models of *Vibrio vulnificus* FJ03-X2 strain with high pathogenicity and strong virulence were established in zebrafish (*Danio rerio*) fibroblast cell line (ZF4 cells) and zebrafish. Zebrafish *tnfb* (*Zetnf-b*) gene was cloned and expressed by *Escherichia coli* BL21 (DE3), and Zetnf-b polyclonal antibody was prepared. Pre-injection of Zetnf-b polyclonal antibody and AG-126 before infecting with *V. vulnificus* could increase the survival rate of zebrafish by 36.6 and 46.7%, respectively. Pre-injection of Zetnf-b polyclonal antibody could effectively decrease the mortality of zebrafish infected by *V. vulnificus*. Thus, TNF polyclonal antibody therapy could be considered as an effective strategy to control *V. vulnificus* in fish.

## Introduction

Fish tumor necrosis factor (TNF) family members are cytokines with multiple effects, which play vital roles in cell growth, differentiation, proliferation and immunity, apoptosis, macrophage activity and autoimmune diseases (1). Fish possess a diversified TNF family. Most of the TNF family members in fish are similar to mammals, but some homologs are unique in fish. However, multiple isoforms of TNF subtypes can be found in several fish species due to a genome duplication event in bony fish (2, 3). TNF-alpha (TNF-α) is one of the important members of the TNF family, which has been identified in a variety of fish species, such as zebrafish (*Danio rerio*) (4), large yellow croaker (*Larimichthys crocea*) (5), Japanese flounder (*Paralichthys olivaceus*) (6) and grouper (*Epinephelus coioides*) (7). In addition, TNF-α has multiple homologs. Two copies of TNF-α have been cloned in orange-spotted grouper (*E. coioides*) (8), rainbow trout (*Oncorhynchus mykiss*) (9), zebrafish and medaka (*Oryzias latipes*) (10). There are two TNF paralogs in zebrafish, including TNF-α and TNF-b genes. Zebrafish TNF-α has been found to play a key role in immune defense. Loss of TNF signaling increases mortality and its susceptibility to *Mycobacterium marinum* (11). It plays a vital role in resisting bacterial (*Aeromonas hydrophila* and *Edwardasiella tarda*) and viral (Spring viremia of carp virus, SVCV) infections (12). Zebrafish TNF-b participates in various immune responses of zebrafish, such as bacterial infections, antibiotic peptides, zebrafish liver development and cold stress response (13–17). However, its role in resisting bacterial infections is rarely studied.

*Vibrio vulnificus* is the main pathogen of important economic fish, such as European eel (*Anguilla Anguilla*), large yellow croaker and grouper. As one of the most serious aquatic pathogens, it can cause hemorrhagic sepsis and the death of aquatic animals. Also, it can infect people through wounds or foodborne pathogenic infections (18, 19). The continuous increase of antibiotics and other chemical drugs (e.g., potassium permanganate, malachite green) has continuously enhanced antibiotic resistance (AMR) of *V. vulnificus* (20). The development of a safe and efficient *V. vulnificus* vaccine or antibiotic substitute is important for the prevention and control of *V. vulnificus* infection.

Therapeutic antibodies can block microbial pathogenesis via different mechanisms, including direct neutralization, agglutination, fixation with activation of complement, activation of effector cells and blockade of adhesion (21, 22). A previous study observed that polyclonal anti-*C. albicans* antibodies could enhance the inhibition of fungi in zebrafish and promote fish survival (23). Polyclonal antibody against the purified recombinant outer membrane protein (OmpU) of *V. alginolyticus* in rabbits was injected into the intraperitoneal cavity to immunize the crimson snapper (*Lutjanus erythropterus*). The result presented that the polyclonal antibody could significantly improve the survival rate of the immunized group after being infected with *V. alginolyticus* (24). Thus, polyclonal antibody may be considered a good choice to fight against *V. vulnificus* infection. Human TNF-α has been intensively studied, and drugs (e.g., TNF-α blockers) were developed in the form of TNFα antibodies to treat many diseases, including osteoporosis, psoriasis, arthritis and Crohn disease. The TNF superfamily is considered as an active target for human drug development (25). However, there is no information on the application of TNF antibody in controlling fish diseases.

Zebrafish is a good model in laboratory research for several features, including small size, ease of raising, high yield, low cost and reliable experimental results. Zebrafish are used in bacterial or viral infection modeling, cytokine immune mechanism, immune evaluation of vaccines and drug discovery or screening (26–28).

This study aimed to investigate the function of zebrafish TNF-b polyclonal antibody in fighting against *V. vulnificus* infection. We investigated the expression of zebrafish TNF-b (*Zetnf-b*) in immune-related tissues of zebrafish in response to the intraperitoneal infection of *V. vulnificus* and that in the infected ZF4 cells at different time points. *V. vulnificus* infection models of ZF4 cells and zebrafish were developed to study the role of *Zetnf-b* during infection. Finally, the *Zetnf-b* gene was cloned, and the effect of its polyclonal antibody on resistance to *V. vulnificus* infection was determined in zebrafish. The results provide a perspective of TNF antibody therapy for the prevention and treatment of *V. vulnificus* in fish.

## Materials and Methods

### Ethics Statement

All zebrafish were anesthetized with tricaine methane sulfonate (MS-222, Sigma, St. Louis, MO, USA), and surgeries were performed. All animal experiments were conducted in accordance with the guidelines and regulations of the Institutional Animal Care and Use Ethics Committee of Fujian Academy of Agricultural Sciences (Permit Number: BI-AEC-2021055006).

### Fish, Sprague Dawley (SD) Rats, Cells and Pathogenic Bacteria

The zebrafish (AB strain) and ZF4 (ATCC CRL-2050) were purchased from the China Zebrafish Resource Center (Wuhan, China). The zebrafish with an average weight of 0.2 g were raised in a flow through pure water aquaculture system. No disease was observed during breeding. The ZF4 cells were maintained at 28°C in DME/F12 medium (Hyclone, Logan, UT, USA) with 10% fetal bovine serum (Gibco, South America) containing 10 U/ml penicillin and 10 μg/ml streptomycin in a 5% CO_2_ cell incubator. Sprague Dawley (SD) rats of Specific-pathogen-free (SPF) grade were purchased from the Laboratory Animal Center of Fujian Academy of Traditional Chinese Medicine, Fuzhou, China. The fish bacterial pathogen, *V. vulnificus* FJ03-X2 strain, which possesses strong virulence and high pathogenicity, was isolated from the sick European eel (*Anguilla anguilla*) and maintained in our laboratory. Its half-lethal dose (LD_50_) for elvers (15–30 g/tail) at a water temperature of 28°C was 2.3–7.1 × 10^3^ colony forming unit (CFU)/tail. Tryptic soy broth (TSB) medium (HKM, Guangdong, China) was used to cultivate *V. vulnificus* FJ03-X2 strain.

### Cloning and Expression Analysis of *Zetnf-b*

*Zetnf-b*-F and *Zetnf-b*-R primers (Table 1) containing restriction enzyme sites for EcoR I at the 5′-terminus and BamH I at the 3′-terminus were designed following the sequence of zebrafish *tnf-b* gene (Accession no.: NM_001024447). Total RNA from liver and kidney of 3 healthy zebrafish was extracted using Trizol Reagent solution (Takara, Beijing, China) according to the manufacturer's protocol. *Zetnf-b* was amplified by reverse transcription-polymerase chain reaction (RT-PCR) and cloned into pMD18-T vector (TaKaRa, Beijing, China). The positive clone plasmid was subjected to double enzyme digestion and sub-cloned into the expression vector pET-32a (Takara, Beijing, China) to get recombinant expression plasmid pET32a–*Zetnfb*. The recombinant proteins of *Zetnfb* (rZetnfb) were obtained by *Escherichia coli* BL21 (DE3) containing pET32a–*Zetnfb* and confirmed by 12% sodium dodecyl sulfate polyacrylamide gel electrophoresis (SDS-PAGE).

**Table 1:** Gene specific primers used for *Zetnf-b* gene amplification and expression analysis [quantitative real-time polymerase chain reaction (qRT-PCR)] in this study.

**Gene**	**Sequence (5′-3′)**
*Zetnf-b*-F^a^	CGGAATTCATGGTGAGATACGAAACAAC
*Zetnf-b*-R^b^	CCCAAGCTTTCACAACGCGAACACCC
β-actin-F^a^	CACTTCACGCCGACTCAAAC
β-actin-R^b^	TCGGGGATGCTTATTTGCCA
*Zetnf-b*-RT^a^	GGCATGTGATGAAGCCAAACGAA
*Zetnf-b*-RT^b^	GCCAACCCATTTCAGCGATTG

a * F, Forward*;

b *R, Reverse*.

### Purification of *rZetnfb* and Preparation of Polyclonal Antibody

The rZetnfb was purified by cutting the target band from the SDS-PAGE gel and quantified. T 10 basic ULTRA-TURRAX ® (IKA, Staufen, Germany) was used to mix the target protein and adjuvant in a volume ratio of 1:1. Complete Freund's adjuvant (Sigma, Germany) was mixed with rZetnfb for the first immunization, and incomplete Freund's adjuvant (Sigma, Germany) was mixed with rZetnfb for the last immunization. Five SD rats of SPF grade were inoculated with the mixed substance through multiple subcutaneous injections to prepare polyclonal antibody. A part of SD rats collected before immunization was used as a negative control in the experiments. All the serum samples of SD rats were collected after the immunization. The titer of the serum polyclonal antibody was determined by enzyme-linked immunosorbent assay (ELISA) according to the method described previously (29). The specificity of the polyclonal antibody was analyzed by western blotting as the method described previously (30).

### Cell Toxicity Determination of *V. vulnificus* FJ03-X2

The ZF4 cells were plated in 6-well culture plates and divided into 4 groups with 3 wells per group. These were incubated overnight in Dulbecco's Modified Eagle Medium/Nutrient Mixture F-12 (DME/F-12) in an incubator with 5% CO_2_ and 95% air at 28°C. *V. vulnificus* FJ03-X2 cells in the logarithmic growth phase were diluted with (TSB) medium at a density of 1.0 × 10^6^ CFU/ml. One group of cells without *V. vulnificus* inoculation was used as a negative control. The other three groups were inoculated with *V. vulnificus* for 12 h at three multiplicity of infection (MOI) of 1:1, 1:10 and 1:100, respectively. Optical density (OD) was measured at 490 nm (OD_490_) with a full-wavelength microplate reader (Bio-Rad, Hercules, CA, USA). The percent cytotoxicity in each plate was calculated through the level of lactate dehydrogenase (LDH) using the standard procedure from CytoTox96 non-radioactive cytotoxicity assay (Promega, Madison, WI, USA). A low control (LC) and a high control (HC) were set up, and the percentage of cytotoxicity was calculated according to following equation:


$$\begin{array}{l} Cytotoxicity=\frac{OD^{V.vulficus}-OD^{LC}}{OD^{HC}-OD^{LC}}\times 100\; \end{array}$$

### LD_50_ Determination

A total of forty zebrafish of similar size (Tail weigh of 0.22 g ± 0.02 g) and good growth condition were divided into 4 groups. According to the weight of the zebrafish, three groups were injected intraperitoneally with 1.4 × 10^5^ CFU/g, 5.61 × 10^4^ CFU/g and 1.4 × 10^4^ CFU/g *V. vulnificus*, respectively. The negative control group was injected with 10 μl of sterilized phosphate-buffered saline (PBS). The zebrafish status and survival rate were observed and recorded once every 3 h until 24 h. The LD_50_ of *V. vulnificus* infection in zebrafish was determined according to the recorded survival rate and the concentration of bacteria for challenging zebrafish.

### Challenge Test

The experimental group was injected intraperitoneally with 10 μl *V. vulnificus* [LD_50_ =4.39 × 10^4^ (CFU/g) per fish]. The control group was injected with 10 μl of sterilized PBS. Kidney, muscle and intestine samples were collected from three fish of each group at different time points [1 h post infection (hpi), 2, 4, 8 and 12 hpi] for RT-PCR detection. Kidney samples from healthy and infected zebrafish at different time points (2, 4, 6, 8, 12 and 24 hpi) were collected for western blotting. The activity and survival rate were recorded.

### Cell or Tissue Samples, RNA Extraction and Quantitative Real-Time Polymerase Chain Reaction (qRT-PCR)

Cell samples for RNA extraction refers to ZF4 cells infected with 0.1 MOI *V. vulnificus* at different time points (0.5, 1, 2, 4, 8 and 12 hpi). Intestinal, muscle and kidney samples were collected from control and experimental groups. The TNF inhibitor AG-126 was purchased from Sigma-Aldrich (St. Louis, MO, USA). RNA extraction was conducted by using Trizol Reagent (Takara, Beijing, China) according to the manufacturer's protocol. qRT-PCR was performed using TB GreenTM Premix Ex TaqTM II reagent Kit (Takara, Beijing, China) with the Step Two TM Real-Time PCR System (Applied Biosystems, Waltham, MA, USA). Primers specific for *Zetnf-b* and β*-actin* are shown in Table 1. The relative mRNA expression level of *Zetnf-b* was calculated using the relative quantification analysis module of the 2^−ΔΔ*Ct*^ method (31) based on Ct values. All qRT-PCR experiments were repeated three times.

### Western Blotting

Kidney samples were prepared as descripted in 2.7 for Western blotting. Briefly, total protein was obtained using the Invent Total Protein Extraction Kit (Invent SD001/SN002, Plymouth, USA), and the protein concentration was detected by BCA protein content detection kit (Bioplatform BP104, Nanjing, China). Samples with equal amounts of protein were subjected to SDS-PAGE and transferred to polyvinylidine difluoride membrane (Millipore, Bedford, MA, USA). After blocking, the membrane was incubated with the primary polyclonal antibody of Zetnf-b (1:1,000), which was prepared as descripted in 2.4 and rabbit polyclonal anti-β-actin (1:1,000; Abcam; UK) at 4°C for 12 h. After washing, the membrane was incubated with goat-anti-mouse (1:8,000; Utibody; Tianjin, China) and Goat Anti-mouse Rabbit (1:8,000; Utibody; Tianjin, China) secondary antibodies for 2 h at 28°C. Detection was performed using an ECL hemiluminescence kit (ThermoFisher, Waltham, MA, USA). After the film was scanned, the gray value of each protein band was measured, and data were presented as a ratio of the value to that for-actin. Zetnf-b expression in the kidney of mock-infected zebrafish was set to 1.0 and marked as 0 h, and the expression of Zetnf-b in infected zebrafish at 2–24 hpi was compared to the expression at 0 h.

### Statistical Analyses

Experiments were performed in triplicate and the results were expressed as mean ± standard error (SE). All data were analyzed using Statistical Package for the Social Sciences (SPSS) for Windows (Version 16.0). The difference between the control group and the experimental group was tested by independent sample *t* test analysis. The marker ^*^ represents *P* < 0.05, which is considered to be statistically significant, and ^**^ represents *P* < 0.01, which is considered to be significantly different.

## Results

### Toxicity Determination in ZF4 Cells and LD_50_ Determination in *V. vulnificus* FJ03-X2 Infected Zebrafish

**Figure 1:**
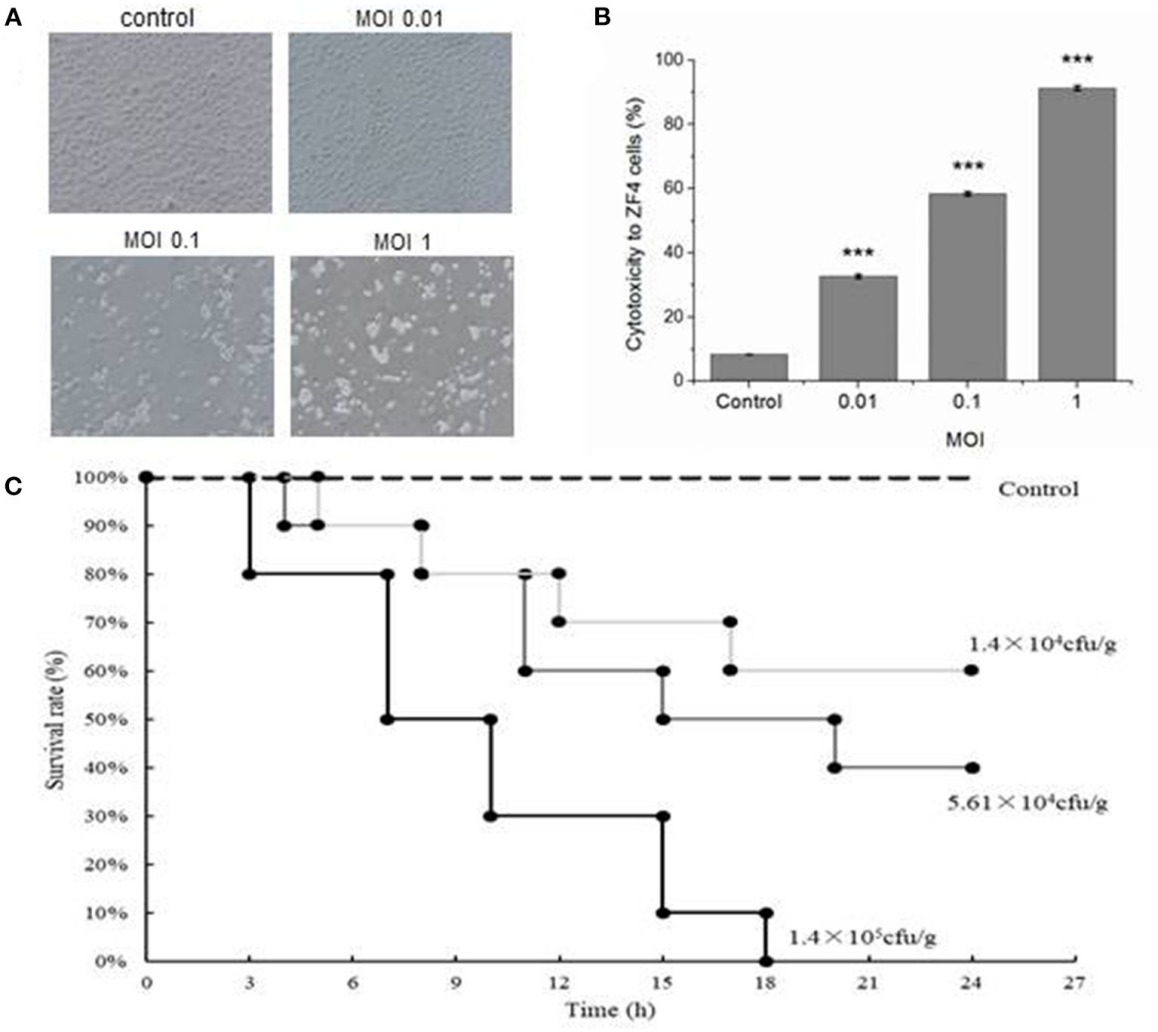
Cytotoxicity to ZF4 cells and lethality to zebrafish infected by *Vibrio vulnificus*. **(A)** The growth status of ZF4 cells under different multiplicity of infection (MOI) conditions. The cells were healthy in the control group; the cells were significantly shrunken when MOI was 0.01; the cells were severely apoptotic when MOI was 0.1; the cells were totally dead when MOI was 1. **(B)** The cytotoxicity to ZF4 cells under different MOI conditions. **(C)** The survival rate of zebrafish infected with different doses of *V. vulnificus*. Data are presented as mean ± standard error (SE) from three independent experiments. ^***^indicates significant difference at *P* < 0.001.

ZF4 cells without *V. vulnificus* infection were elongated, and these were in a healthy cell growth state with high fusion and adherence. Cells in the first group (MOI = 0.01) shrank significantly. Most of the cells in the third group (MOI = 1) lost their adhesion, and the cell structure was broken (Figure 1A). The cytotoxic effect of *V. vulnificus* determined by evaluation LDH activity released from ZF4 cells is shown in Table 2 and Figure 1B. All the results verified the strong virulence of *V. vulnificus* FJ03-X2.

**Table 2:** Cytotoxic effect of *Vibrio vulnificus* determined by evaluation of lactate dehydrogenase (LDH) activity released from ZF4 cells.

**Cell culture**	**Control**	**MOI = 0.01**	**MOI = 0.1**	**MOI = 1**
Cytotoxicity %	7.76% ± 0.021	32.7% ± 0.048	58.4% ± 0.036	91.3% ± 0.031

Zebrafish in the control group injected with PBS were all alive and maintained in a healthy condition during the experiment. The mortality rates were 100, 60 and 40% in 24 h in infection groups with 1.4 × 10^5^, 5.61 × 10^4^ and 1.4 × 10^4^ CFU/g of *V. vulnificus*, respectively (Figure 1C). The LD_50_ of *V. vulnificus* challenged zebrafish at 24 h was 4.39 × 10^4^ CFU/g.

### Expression Analysis of *Zetnf-b* in ZF4 Cells and Zebrafish With *V. vulnificus* Infection

**Figure 2:**
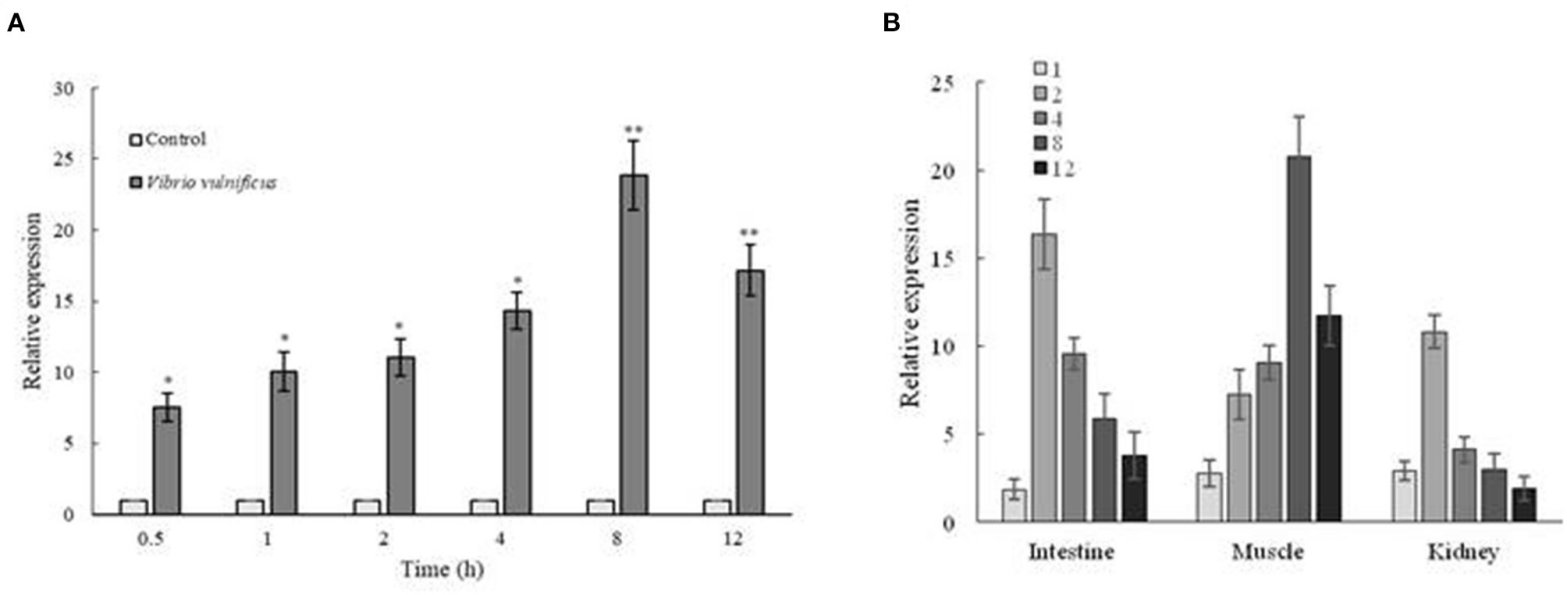
Expression analysis of *Zetnf-b* in ZF4 cells and zebrafish both infected by *Vibrio vulnificus*. **(A)** Expression profile of *Zetnf-b* using qRT-PCR in ZF4 cells infected with 0.1 MOI *V. vulnificus*. **(B)** Tissue expression of *Zetnf-b* using qRT-PCR in zebrafish infected with *V. vulnificus*. Total RNA was extracted from the cells, and qRT-PCR was conducted to analyze Ze*tnf-b* and β*-actin* gene expression at 1 h post-stimulation (1 hpi), 2, 4, 8 and 12 hpi. Data are presented as mean ± standard error (SE) from three independent experiments. ^*^ indicates significant difference at *P* < 0.05. ^**^ indicates significant difference at *P* < 0.01.

The expression of *Zetnf-b* in ZF4 cells was significantly up-regulated after *V. vulnificus* infection at 0.5 hpi compared with the control group (Figure 2A). The *Zetnf-b* expression was increased with time between 1 and 8 h, and the peak was observed at 8 h. Although the expression began to drop at 12 hpi, it was significantly higher than the control group.

As shown in Figure 2B, the *Zetnf-b* expression in the intestine and kidney was increased with time after infection. The expression level in the intestinal and kidney reached the peak at 2 hpi, which was up-regulated by 16.36-fold and 10.81-fold higher than the control group, respectively. The expression of *Zetnf-b* in muscle was also increased with time and its peak was observed at 8 h, which was increased by 20.80-fold. These results indicated that *V. vulnificus* infection could cause the up-regulation of *Zetnf-b* in the main immune organs of zebrafish.

**Figure 3:**
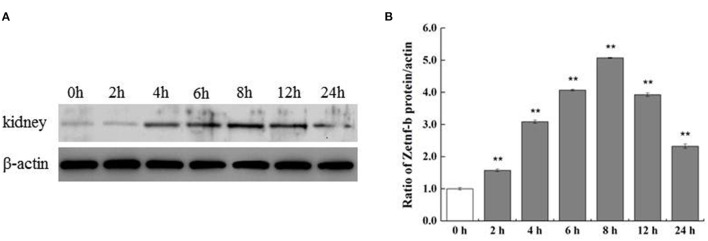
Western blotting of Zetnf-b protein in kidney of zebrafish infected by *V. vulnificus* at different time points. **(A)** Western blotting of Zetnf-b protein in the kidney of zebrafish infected by *V. vulnificus* at different time points. Kidney of mock-infected zebrafish was marked as “0 h” and used as the control. Polyclonal antibody of Zetnf-b (1:1,000) and rabbit polyclonal anti-β-actin (1:1,000) were used as the primary antibody. **(B)** The ratio of Zetnf-b protein/actin. Data are presented as mean ± standard error (SE) from three independent experiments. ^**^ Indicate significant difference at ^**^P < 0.01.

To further verify the expression of *Zetnf-b* in zebrafish response to *V. vulnificus* infection, Zetnf-b polyclonal antibody was developed in 3.3, and western blot assay was performed to determine the protein expression level of Zetnf-b. As shown in Figures 3A,B, compared to the healthy zebrafish (0 h), the expression of Zetnf-b gradually increased and reached a maximum value at 8 hpi. Until 24 h after infection, the expression of Zetnf-b was higher than that of the control. The data of western blotting and qRT-PCR indicated that a TNF inflammation storm erupted after zebrafish were attacked by *V. vulnificus*.

### ELISA and Western Blotting of Zetnf-b Polyclonal Antibody

**Figure 4:**
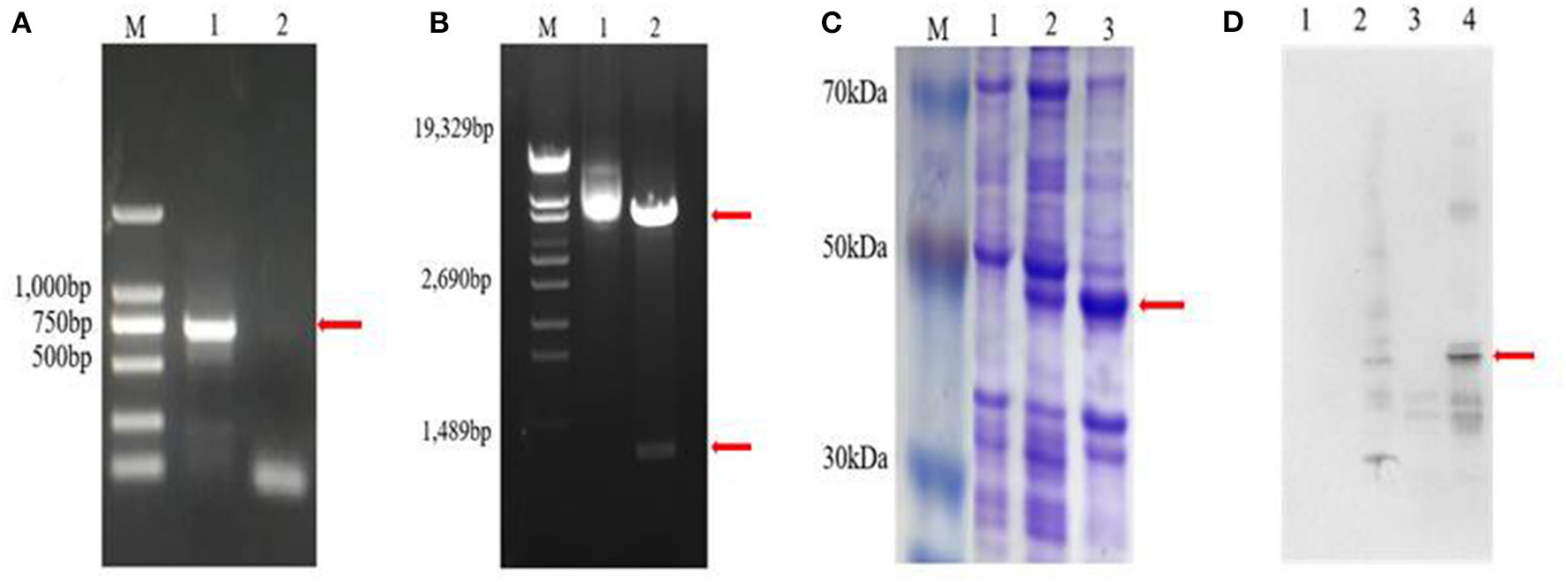
Cloning and expression of *Zetnf-b*. **(A)** Polymerase chain reaction (PCR) amplification of *Zetnf-b* (M: DL2000 marker. 1: complementary DNA (cDNA) template. 2: sterile water negative control). **(B)** Double digestion results of recombinant plasmid pET32a-*Zetnfb* (M: λ-EcoT14 I digest. 1: recombinant plasmid pET32a-*Zetnfb*. 2: recombinant plasmid pET32a-*Zetnfb* double digestion [upper 5900 bp, lower 729 bp)]. **(C)** Expression of rZetnfb (M: low molecular weight pre-stained marker. 1: pET32a-*Zetnfb*; not induced. 2: pET32a-*Zetnfb* induced supernatant. 3: pET32a-*Zetnfb* induced precipitation). **(D)** Western blotting of *Zetnf-b* polyclonal antibody (1: pET32a; no-load not induced. 2: pET32a; no-load induced. 3: pET32a-*Zetnfb*; not induced. 4: pET32a-*Zetnfb*; induced).

*Zetnf-b* gene (729 bp) was cloned (Figure 4A). Also, the prokaryotic expression vector pET32a was formed (Figure 4B). As shown in Figure 4C, a fusion protein with a molecular weight of approximately 40 kDa was induced to express in an insoluble form.

Indirect ELISA was performed by coating 96-well ELISA plates with purified rZetnfb protein as an antigen to detect the titer of polyclonal serum. The absorbance value of the positive serum was 2.78 times higher than that of the pre-immune rat serum at a dilution of 1:160,000 (Table 3). This showed the rZetnfb polyclonal antibody had a high titer up to 1:160,000. Then, western blotting was used to detect the specificity of rZetnfb polyclonal antibody. The result showed a specific band (40 kDa) in the induced expression group, while no specific binding band was observed in the empty control (Figure 4D), indicating that the polyclonal antibody showed strong specificity to rZetnfb.

**Table 3:** Enzyme-linked immunosorbent assay (ELISA) determination of rZetnfb polyclonal antibody titer.

**Dilution**	**Positive serum OD_**492**_:Negative serum OD_**492**_**
1:20,000	8.81
1:40,000	6.15
1:80,000	4.17
1:160,000	2.78
1:320,000	1.75

### Pre-injection of Zetnf-b Polyclonal Antibody Improved the Survival Rate of *V. vulnificus* Infected Zebrafish

**Figure 5:**
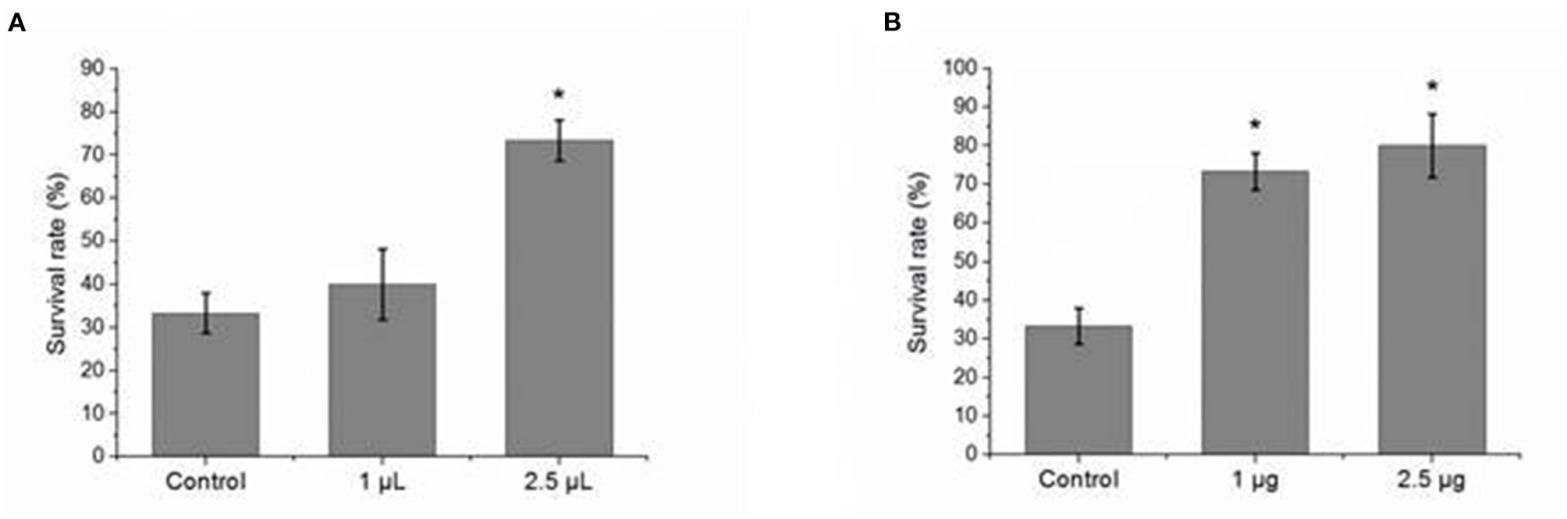
Effect of pre-injected Zetnf-b polyclonal antibody or AG-126 to zebrafish before challenging with *Vibrio vulnificus*. **(A)** The effect of pre-injection of a certain dose of Zetnf-b polyclonal antibody on the survival rate before challenging with *V. vulnificus*, using pre-immune rat serum as control. The survival rate was 73.3% at the dose of 2.5 μl/tail. **(B)** The effect of pre-injection of a certain dose of AG-126 on the survival rate before challenging with *V. vulnificus*, using phosphate-buffered saline (PBS) as control. The survival rate was 80% at the dose of 2.5 μg/tail. Zebrafish survival rate was calculated according to the formula. Survival rate %=(Survived zebrafish / Total fish) × 100%. Data are presented as mean ± standard error (SE) from three independent experiments. ^*^ Indicates significant difference at *P* < 0.05.

To study the effect of Zetnf-b polyclonal antibody on *V. vulnificus* infection, the zebrafish were pre-injected intraperitoneally with different doses of Zetnf-b polyclonal antibody following *V. vulnificus* infection. Pre-immune rat serum and PBS were pre-injected as controls for the antibody and inhibitor groups, respectively. The survival rates of zebrafish pre-injected with 2.5 μl/tail of Zetnf-b polyclonal antibody and TNF inhibitor AG-126 reached up to 73.3 and 80.0%, respectively, while the survival rates of zebrafish in the antibody and inhibitor control groups were 33.3 and 36.7%, respectively (Figures 5A,B). The above results showed that pre-injection with Zetnf-b polyclonal antibody could significantly reduce the mortality of infected zebrafish. The results indicated that Zetnf-b polyclonal antibody effectively reduced inflammation and resisted *V. vulnificus* infection in zebrafish.

## Discussion

Cell injury is an important factor in the pathogenesis of *V. vulnificus*. Virulence factors of *V. vulnificus*, such as cytolysin, repeats-in-toxin A1 (RtxAl) and extracellular protease (ECPase), can cause cell injury (32–34). In this study, ZF4 cells infected by *V. vulnificus* shrunk quickly and significantly, and lost their adhesion. Their cell structures were broken, which is due to the virulence factors released by *V. vulnificus*. In the zebrafish infection model, *V. vulnificus* demonstrated an acute infection to zebrafish. More than half of the zebrafish died due to acute sepsis within 12 h, and the mortality was dependent on the quantity of *V. vulnificus*. The LD_50_ of *V. vulnificus* to zebrafish at 24 h was 4.39 × 10^4^ CFU/g. These data verified that the *V. vulnificus* FJ03-X2 was a highly pathogenic and virulent strain. Therefore, it is significant to choose this strain to establish a zebrafish infection and immune model, and it helps for targeting the suitable strain to develop the effective vaccine of *V. vulnificus* and provide a reference for other *Vibrio* spp. infection models.

Endotoxins and exotoxins of bacterial are the most potent inducers of inflammatory cytokines. Sepsis is associated with a cytokine storm since TNF was first detected the bloodstream of patients with meningococcal infection in 1986, followed by the discovery of interleukin-1 (IL-1), IL-6, IL-8, and IL-10 (35). Sepsis is one of the typical symptoms in organisms caused by toxins of *V. vulnificus*. We also detected a Zetnf-b strom in *V. vulnificus* infection *in vitro and vivo*. *In vitro*, the expression of *Zetnf-b* was increased rapidly after *V. vulnificus* infection. The result is similar to the result of a previous study, in which the expression of immune response genes of ZF4 cells could be significantly induced after *A. hydrophila* NJ-1 infection (36). *In vivo, V. vulnificus* infection also can quickly induce the up-regulation of *Zetnf-b* in tissues, including intestine, kidney and muscle. The western blotting in the kidney showed a result of a consistent trend with qRT-PCR. In previous reports, the expression of *TNF-*α in liver and kidney of turbot (*Psetta maxima*) was significantly up-regulated after stimulating by *V. pelagius* (37). In zebrafish infected with *V. parahaemoglobin, TNF-*α in their spleen increased significantly in a time-dependent manner, and reached a peak at the 12 h after infection and then declined (38). Moreover, the TNF-α, IL-1β and IFN-γ genes were significantly up-regulated in Japanese flounder injected by *V. anguillarum* (39). Our study confirmed that *V. vulnificus* infection could strongly trigger a TNF-b cytokine storm in cells and zebrafish.

However, the overwhelming production of inflammatory cytokines can lead to organ dysfunction and eventually death. To confirm the function of cytokine Zetnf-b in fighting against *V. vulnificus* infection, Zetnf-b polyclonal antibody or TNF inhibitor AG-126 was pre-injected to zebrafish. We found that pre-injection with Zetnf-b polyclonal antibody significantly increased the survival rate of *V. vulnificus* infected zebrafish. Figure 4B showed that the survival rates of zebrafish pre-injected with 2.5 μl/tail of Zetnf-b polyclonal antibody and the control groups were 73.3% and 36.7%, respectively. The Zetnf-b polyclonal antibody neutralizes the Zetnf-b factor and alleviates the inflammation. In the AG-126 group, the survival rate was 80% at the dose of 2.5 μg/tail (Figure 5A). The tyrosine kinase phosphorylation inhibitor AG-126, could block the release of TNF in the TNF signaling pathway, reducing the formation of TNF. AG-126 reduced TNF in cytokine storm in an inflammatory response. The above results indicated that Zetnf-b polyclonal antibody had a positive effect as AG-126 against *V. vulnificus* infection in zebrafish. In addition, it confirmed that pre-injection of Zetnf-b polyclonal antibody aimed to inhibit TNF release or TNF activity, thereby reducing the inflammatory cytokine storm caused by bacterial infection and decreasing fish mortality, which is an effective attempt to treat bacterial infection in zebrafish.

In conclusion, our study demonstrates that *Zetnf-b* plays an important role in inflammatory storms induced by *V. vulnificus* infection. Pre-treatment of Zetnf-b polyclonal antibody and TNF inhibitor AG126 can help zebrafish to resist bacterial infections and improve the survival of zebrafish. This study provides a useful reference for the study of the biological characteristics of TNF, especially TNF-b, in important aquaculture fish and presents the TNF antibody therapy for the prevention and treatment of the *V. vulnificus* in the aquaculture industry.

## Data Availability Statement

The datasets presented in this study can be found in online repositories. The names of the repository/repositories and accession number can be found below: accession no.: NM_001024447.

## Author Contributions

XC and CL were responsible for the conception of the study. SL performed the cloning, expression, protein purification of zebrafish TNFb, performed data interpretation and wrote the manuscript. CJ performed the *V. vulnificus* infection model in ZF4 cells and zebrafish, and also prepared the TNFb polyclonal antibody. HC was responsible for ZF4 cell and *V. vulnificus* culture. LZ and LK performed the experiment of pre-injecting the TNFb polyclonal antibody in zebrafish before *V. vulnificus* infection. CL reviewed the manuscript and provided critical suggestions. All authors discussed the results and approved the final manuscript for publication.

## Funding

This work was funded by the Fujian public welfare research project (2019R1027-5). Meanwhile, this work was supported by the National Nature Science Foundation of China (31300766, 31100658), Innovation Team Projects of Fujian Academy of Agricultural Science (STIT2017-03), Special Fund for Public-interest Scientific Institutions of Science and Technology Plan Projects of Fujian Province (2018R1019-7), and Provincial special support for high-level talents Double Hundred Plan special project.

## Conflict of Interest

The authors declare that the research was conducted in the absence of any commercial or financial relationships that could be construed as a potential conflict of interest.

## Publisher's Note

All claims expressed in this article are solely those of the authors and do not necessarily represent those of their affiliated organizations, or those of the publisher, the editors and the reviewers. Any product that may be evaluated in this article, or claim that may be made by its manufacturer, is not guaranteed or endorsed by the publisher.
